# Periostin promotes tumor angiogenesis in pancreatic cancer via Erk/VEGF signaling

**DOI:** 10.18632/oncotarget.9512

**Published:** 2016-05-20

**Authors:** Yang Liu, Fan Li, Feng Gao, Lingxi Xing, Peng Qin, Xingxin Liang, Jiajie Zhang, Xiaohui Qiao, Lizhou Lin, Qian Zhao, Lianfang Du

**Affiliations:** ^1^ Department of Ultrasound, Shanghai General Hospital, Shanghai Jiaotong University School of Medicine, Shanghai 200080, China; ^2^ Department of Instrument Science and Engineering, Shanghai Jiao Tong University, Shanghai 200240, China; ^3^ Department of Pathophysiology, Key Laboratory of Cell Differentiation and Apoptosis and National Ministry of Education, Shanghai Jiaotong University School of Medicine, Shanghai 200025, China

**Keywords:** periostin, pancreatic cancer, angiogenesis, Erk, VEGF

## Abstract

Pancreatic cancer (PaC) consists of a bulk of stroma cells which contribute to tumor progression by releasing angiogenic factors. Recent studies have found that periostin (POSTN) is closely associate with the metastatic potential and prognosis of PaC. The purpose of this study is to determine the role of POSTN in tumor angiogenesis and explore the precise mechanisms. In this study, we used lentiviral shRNA and human recombinant POSTN protein (rPOSTN) to negatively and positively regulate POSTN expression *in vitro*. We found that increased POSTN expression promoted the tubule formation dependent on human umbilical vein endothelial cells (HUVECs). Moreover, knockdown of POSTN in PaC cells reduced tumor growth and VEGF expression *in vivo*. In accordance with these observations, we found that Erk phosphorylation and its downstream VEGF expression were upregulated achieved in rPOSTN-treated groups, opposing results were obversed in POSTN-slienced group. Meanwhile, Erk inhibitor SCH772984 significantly decreased VEGF expression as well as tubule formation of HUVECs in rPOSTN-treated PaC cells. Taken together, these findings suggest that POSTN promotes tumor angiogenesis via Erk/VEGF signaling in PaC and POSTN may be a new target for cancer anti-vascular treatment.

## INTRODUCTION

Pancreatic cancer (PaC) is a highly malignant tumor with an overall 5-year survival rate of less than 5% [1, 2]. Therapeutic methods for these patients are scarce, curative resection remains the only potentially therapy for PaC patients until recently. However, patients often present with advanced stage with distant metastases, limiting the chance of surgical resection [3]. Thus, there is a desperate need for the development of new diagnostic biomarkers as well as innovative treatment strategies to improve this situation [4].

PaC is characterized by a prominent desmoplastic reaction and the stroma provides structural support for PaC progression [5, 6]. Therefore, the fibrotic matrix was no longer merely considered as a host barrier against tumor progression, it has now become obvious that it can initiate and promote tumorigenesis [1, 7]. Meanwhile, the stroma is a dynamic cellular microenvironment that is mainly made up of pancreatic stellate cells (PSCs). The typical feature of PaC desmoplastic reaction is quiescent PSCs transform into activated PSCs. Therefore, PSCs are considered to be critical for PaC desmoplastic response. Periostin (POSTN), a 90-kilodalton secretory protein, has been involved in a number of human cancers including PaC. PSCs are the only source of POSTN in PaC. Besides, POSTN expression has been shown to be upregulated 42-fold in PaC compared with normal pancreas at the mRNA level [8]. Once stimulated by tumor cells, PSCs will perpetually be activated and produce excessive extracellular matrix to infiltrate and envelop the normal parenchyma via an autocrine POSTN loop, creating a tumor-supportive microenvironment [1, 5]. In a word, PaC is one of the most malignant cancers and result in a heavy health burden worldwide, new methods are urgently needed to treat this malignancy [9, 10].

Solid tumor growth depends on the vascular vessels to supply nutrients. In general, tumor progression is accompanied by rich angiogenesis to satisfy the metabolic requirements [11]. Tumor metastasis is a major contributor to cancer-related death and the process of capillary formation in tumor is a crucial aspect to enable metastasis, especially when tumors diameter approximates 2 mm [12]. The poor outcome of PaC is closely associate with the tendency to spread to surrounding tissues, approximately 50% of PaC patients present with distant metastatic disease [13], which is significantly correlated with the phenomenon of angiogenesis [14, 15]. Therefore, tumor angiogenesis correlated significantly with metastasis, poor prognosis as well as a poor 5-year overall survival. Many efforts have been made to inhibit the angiogenesis for PaC. However, traditional anti-vascular drug may promote tumor progression by inducing hypoxia which insteadly stimulates angiogenesis, so novel anti-vascular therapy should be applied [12]. To our knowledge, there are various molecular factors involved in vascular growth. Among these, the extracellular signal-regulated kinase (Erk) and its downstream vascular endothelial growth factor (VEGF) signaling pathway have critical roles [16–18]. In particular, VEGF is probably the most important tissue factor and a key angiogenic growth factor that responsible for angioblast differentiation and tube formation [17]. Thus, VEGF overexpression not only considered as a diagnostic marker, but also a poor prognostic factor of PaC.

Technical assessment on tumor angiogenesis over the past decade has focused on contrast-enhanced ultrasound (CEUS) imaging. It is a non-invasive approach to monitor the tumor angiogenesis in real-time with high spatial and temporal resolution. Sonovue is the second-generation ultrasound contrast agent containing stabilized microbubbles of sulfur hexafluoride gas, which exhaled via lung within a few minutes and not impaire the renal function [19]. Thus, CEUS is a feasible and safety way to assess the tumor angiogenesis quantitatively.

In this paper, we elucidate a new finding that sliencing POSTN expression disclosed anti-angiogenesis and anti-metastasis efficacies in PaC. Although POSTN expression has been identified in some other tumors, little is known about its biological functions on PaC, especially on POSTN-related angiogenic mechanism. The aim of this study is to clarify the role of POSTN in angiogenesis in PaC and to explore its potential signaling targets, such as Erk/VEGF signaling pathway. Our new findings will facilitate the understanding of POSTN-mediated angiogenesis mechanisms on PaC.

## RESULTS

### POSTN promotes tubule formation of HUVECs

**Figure 1:**
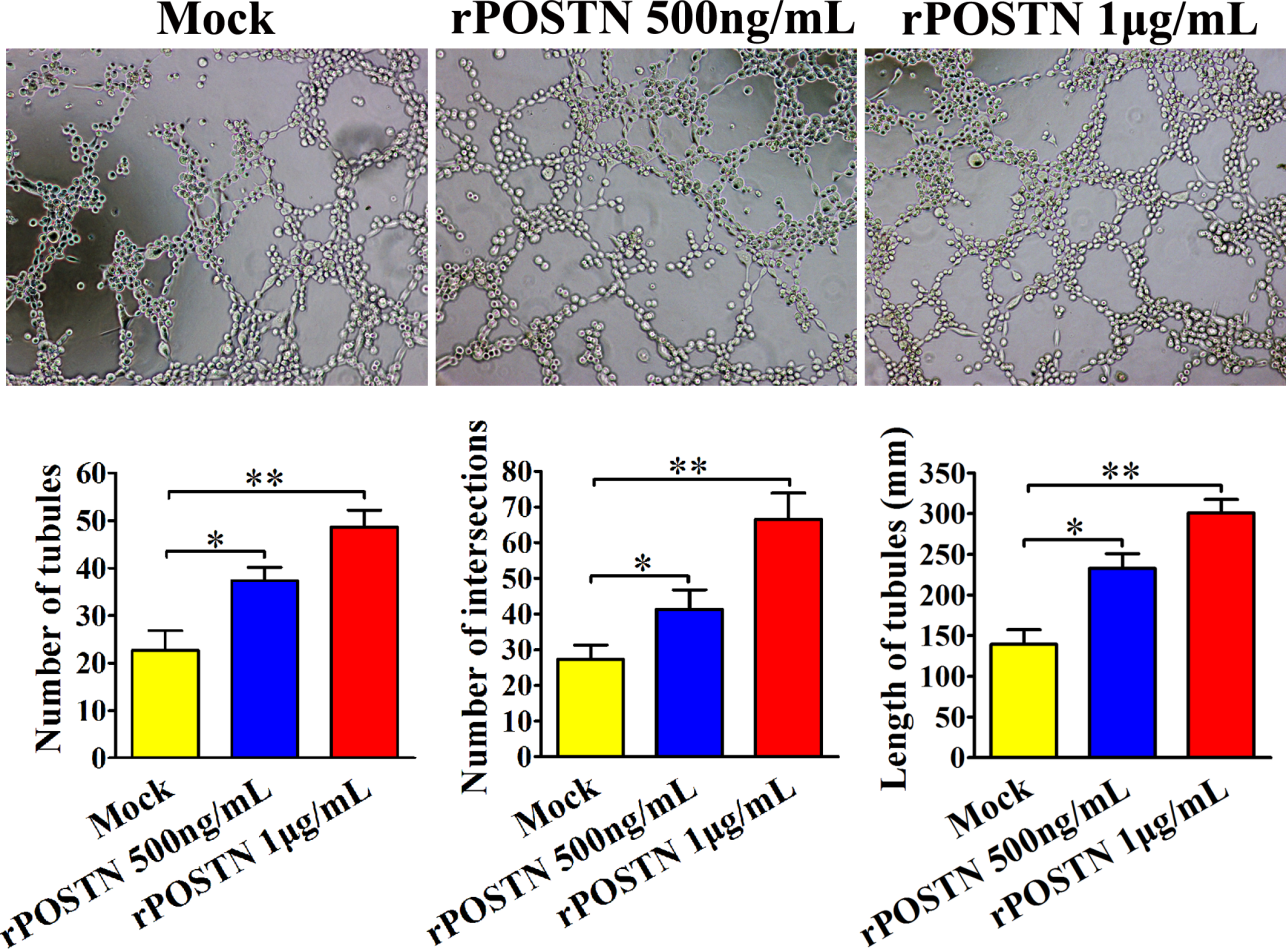
Effect of POSTN on tubule formation *in vitro* In the HUVEC assay, abundant tubules were observed in the rPOSTN-treated group compared with the control group (×200 magnification; Zeiss). Bar charts show numbers of tubules, numbers of nodule intersections, and length of tubules between different groups. *P<0.05 and **P<0.01 vs. Mock.

Ten thousand Human umbilical vein endothelial cells (HUVECs) were suspended in medium with different concentrations of rPOSTN and plated into 96-well plate coated with matrigel. After 8 h incubation at 37°C, tubule formation of each group was assessed under light microscope. We observed increased tubule formation in the rPOSTN-treated HUVECs group whatever in tubular number, tubular length and tubular intersecting nods, compared with the control group. (Figure 1).

### POSTN promotes proliferation, migration and invasion of HUVECs

**Figure 2:**
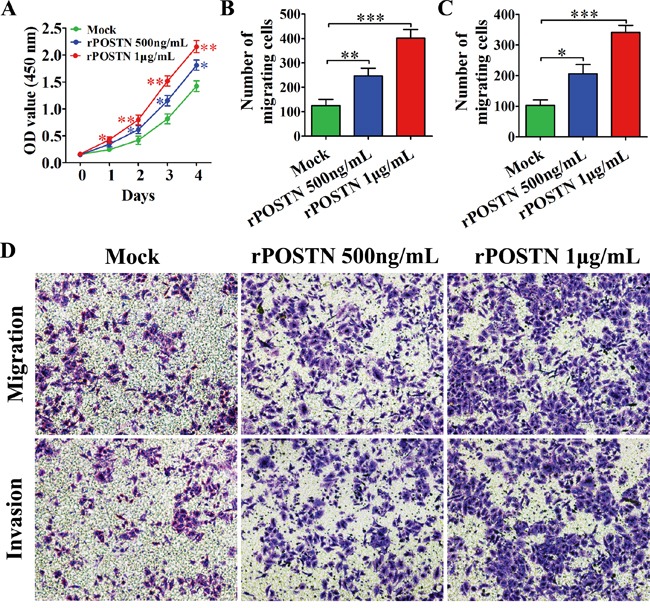
Effects of POSTN on proliferation, migration and invasion of HUVECs **A.** The proliferation of HUVECs was increased in rPOSTN-treated groups compared with the control group. **B.** Histograms showed the numbers of migration cells. **C.** Histograms showed the numbers of invasion cells. **D.** POSTN promoted the migration and invasion of HUVECs (×200 magnification; Zeiss). Data are represented as mean ± SD of three independent experiments.

The tubule formation of HUVECs was associated with HUVECs proliferation, migration and invasion. We observed HUVECs proliferation was significantly increased in rPOSTN-treated group compared with control groups (Figure 2A). Likewise, a similar tendency was observed in migration and invasion. The migration and invasion ability of HUVECs was enhanced in rPOSTN-treated group compared with control groups (Figure 2B, 2C and 2D). Together, our results suggest that POSTN promoted tumor angiogenesis in PaC by stimulating the proliferation, migration and invasion of HUVECs.

### POSTN promotes tumorigenesis *in vivo*

**Figure 3:**
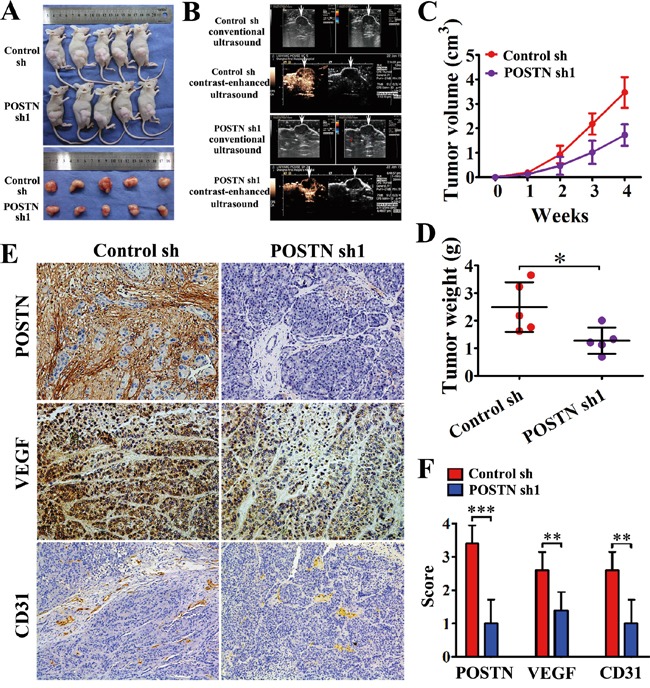
POSTN enhances the tumorigenicity and angiogenesis of pancreatic cancer cells *in vivo* **A.** SW1990 cells were co-injected with control shRNA-transfected PSCs (Control sh) or POSTN shRNA1-transfected PSCs (POSTN sh1) into the right side of nude mice. After 4 weeks the mice were sacrificed. POSTN knockdown group exhibited slower growth than control group. **B.** Conventional ultrasound and contrast-enhanced ultrasound (CEUS) were performed before the mice were sacrificed to evaluate angiogenesis in the xenografts. Fewer blood vessels were observed in the POSTN knockdown group compared with the control group. **C** and **D.** Silencing of POSTN exhibited reduced tumor volume and weight of xenografts. **E.** Immunohistochemical staining showed POSTN deposits in the stroma of xenografts. Xenograft tumors from the POSTN-shRNA group were decreased in VEGF expression and contained significantly fewer CD31-positive small capillary-like vessels than those from the control group (n=15, five random fields). **F.** The statistical analysis on immunohistochemical staining by scores.

The effect of POSTN on tumorigenesis was examined by subcutaneous co-injection of SW1990 and POSTN-sh1 transfected PSCs. We observed that knockdown of POSTN in PSCs inhibited tumor growth compared with the control group (Figure 3A). The mean tumor volume was much smaller in POSTN-silenced group than that in control group (Figure 3C). Besides, the tumor weight was decreased in POSTN-silenced group compared with the control group (2.58±0.21 g vs 1.31±0.19 g, P<0.05) (Figure 3D). Conventional ultrasound and contrast-enhanced ultrasound (CEUS) were performed to evaluate the vessels formation before the mice were sacrificed (Figure 3B). The results showed reduced blood flow signals were observed in POSTN-silenced group compared with the control group. Thus, enough nutrition from increasing vessels regulated by POSTN may be the interpretation about the above findings.

Immunohistochemical staining showed VEGF expression decreased in POSTN-silenced group and significantly fewer CD31 positive capillary-like vessels in POSTN-sh1 xenograft tumors compared with the control group (Figure 3E and 3F). This tendency was congruity with CEUS and consistent with the results *in vitro*. These data collectively indicate that POSTN promoted PaC growth via stimulating vessels formation *in vivo*.

### POSTN upregulates phosphorylated Erk in PaC

**Figure 4:**
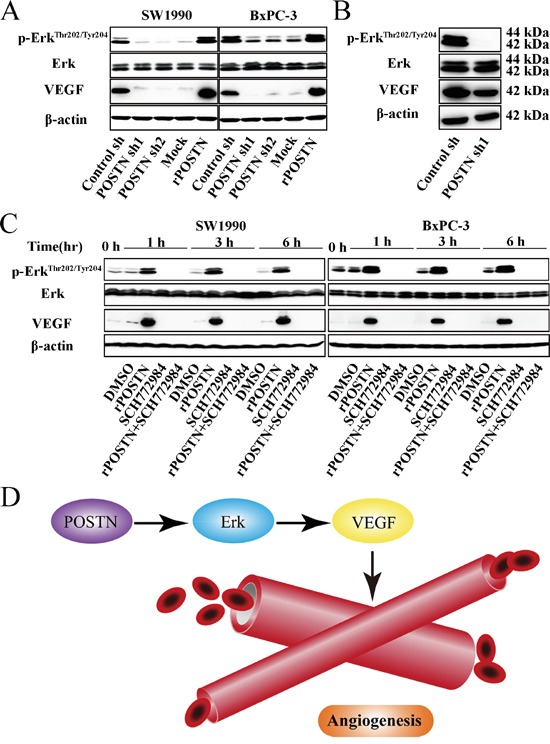
POSTN activates Erk/VEGF signaling to regulate the angiogenesis of pancreatic cancer cells **A.** SW1990 and BxPC-3 cells were treated with the supernatant of control shRNA-transfected PSCs (Control sh), POSTN shRNA1-transfected PSCs (POSTN sh1), POSTN shRNA2-transfected PSCs (POSTN sh2), or human recombinant protein (rPOSTN, 1 μg/mL). After 12 h, cells were harvested and the basal expression of Erk and its downstream molecule VEGF was determined by western blotting. **B.** Xenograft tumors of nude mice from the control-shRNA group and POSTN-shRNA1 group were also subjected to western blotting using the indicated antibodies. **C.** SW1990 and BxPC-3 cells were treated with DMSO, rPOSTN, Erk inhibitor (SCH772984, 20 μM), or rPOSTN plus SCH772984. Cells were harvested after 0, 1, 3, and 6 h, and the basal expression of Erk and its downstream molecule was determined by western blotting. **D.** POSTN activates Erk-VEGF signaling to regulate the angiogenesis of pancreatic cancer cells.

**Figure 5:**
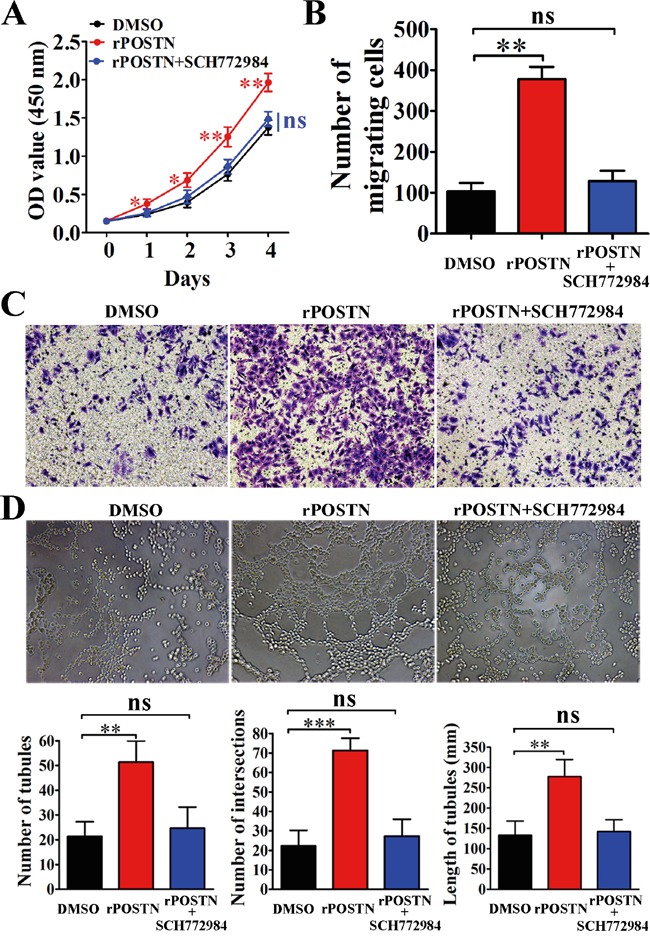
The effects of Erk inhibitor on pancreatic cancer cells **A.** Proliferation of HUVECs was increased by treated with rPOSTN (1 μg/mL) and was completely inhibited by SCH772984. **B.** Histograms showed the numbers of migration cells. **C.** Migration of HUVECs was significantly reduced by treatment with SCH772984. **D.** Tubule formation ability of HUVECs was completely inhibited by SCH772984. Bar charts show numbers of tubules, numbers of nodule intersections, and length of tubules between different groups. *P<0.05, **P<0.01 and ***P<0.001vs. DMSO.

Erk signaling is not only related with tumor angiogenesis, but also tumor metastasis. In this study, POSTN increased Thr202 and Tyr204 phosphorylation of Erk and VEGF expression. Opposing results were observed in POSTN-silenced group compared with the control groups (Figure 4A). Meanwhile, we found Erk phosphorylation and VEGF expression were decreased in POSTN-sh1 xenograft tumor (Figure 4B). As shown in Figure 4C, the effect of POSTN on Erk phosphorylation was completely abrogated when cells were treated with Erk inhibitor SCH772984. Moreover, the phosphorylation of downstream molecule VEGF was correspondingly inhibited. Next, we examined the effects of SCH772984 on cell function and it is worth mentioning that the proliferation, migration and tubule formation of HUVECs were decreased after treated with Erk inhibitor SCH772984 (Figure 5A, 5B, 5C and 5D). Together, these findings suggested that POSTN promoted tumor angiogenesis mainly via Erk/VEGF signaling.

### VEGF in PaC tissues was positively associated with POSTN expression

**Figure 6:**
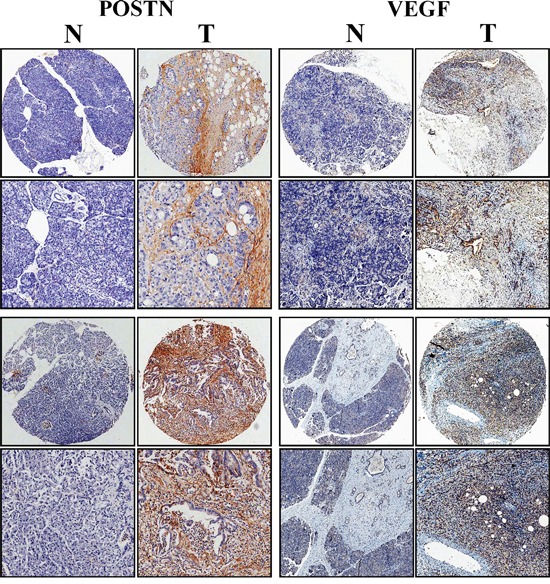
VEGF in pancreatic cancer tissues was significantly correlated with POSTN expression Immunohistochemical staining of the same pancreatic cancer and matched normal tissue with anti-POSTN and anti-VEGF antibody simultaneously. Representative photomicrographs of POSTN and VEGF in pancreatic specimens showed VEGF immunostaining was significantly associated with POSTN expression (magnification, ×50, ×200).

To further investigate the correlation between POSTN expression and VEGF expression, we used TMAs to study POSTN and VEGF expression levels in PaC and corresponding paired normal tissues. We performed immunohistochemical staining for POSTN and VEGF on a large cohort of primary PaC patients simultaneously (n=30). Semiquantitative analysis showed an increased intensity of POSTN and VEGF staining in PaC compared with normal tissues. Both VEGF and POSTN had similar expression pattern in PaC tissues. Besides, POSTN expression was positively associated with VEGF expression in the same patient tissue (Figure 6, Table 1). Positive correlation was found between the expression of these two proteins (r=0.472, P<0.01).

**Table 1:** Expression of POSTN and VEGF in pancreatic cancer tissues

		VEGF		P
		−	+	
POSTN	−	8	2	<0.05
	**+**	6	14	

## DISCUSSION

The microenvironment of cancer is complicated and can maintain stable survival advantages of PaC cells despite various perturbations [20, 21]. When the environment is not optimal, PaC cells can mediate adaptive changes such as stimulation of tumor angiogenesis. Angiogenesis plays a critical role in tumor initiation, progression and metastasis [22], and now it has been considered to be one of the major hallmarks of cancer [23–25]. Tumors require blood supply for nutrition, growth as well as distant metastasis. More and more attention has been focused on tumor angiogenesis, which is significantly correlated with cancer metastasis. The importance of angiogenesis in PaC progression cannot be ignored [26–28]. Surgical resection is a reasonable therapy to PaC patients, patients who have no distant metastasis should undergo surgical resection [29]. However, over 80 ~ 90% patients are diagnosed at the advanced stage, which attributed to the early metastasis and dissemination of the PaC cells [3]. POSTN, originally designated osteoblast-specific factor 2, was found to be overexpressed in various types of human tumors [30, 31], including PaC [1, 32]. POSTN mRNA elevated 42-fold in PaC, and patients with increased expression had a shorter survival tendence [1]. Thus, cancer metastasis is significantly correlated with tumor angiogenesis. However, the detailed tumor-associated angiogenesis function of POSTN in PaC remain to be explored. Based on these observations, we aim to determine the role of POSTN in mediating tumor angiogenesis of PaC and its potential clinical significance. The endothelial tube formation assay is based on the ability of endothelial cells to form 3D tubular structures when coated with a gel of basement membrane extract.

There is plenty of evidence to support the significance of angiogenesis in tumor development. For instance, Zhang *et al.* found that upregulate of POSTN expression may promote angiogenesis in keloids and concluded that POSTN may be a key factor in keloid development [33]. Wang *et al.* reported that high expression of POSTN is closely associated with tumor angiogenesis, progression and poor survival of esophageal squamous cell carcinoma (ESCC) [25]. Hu *et al*. examined POSTN expression in osteosarcoma and explored the relationship of POSTN expression with tumor angiogenesis. They showed that high expression of POSTN significantly correlated with VEGF expression and microvessal density compared to the normal bone tissues [34]. Our experiments also confirmed that rPOSTN significantly promoted angiogenesis in PaC. We verified that POSTN promoted tubule formation dependents on HUVECs and POSTN was also able to accelerate HUVECs proliferation, migration and invasion, which contributed to tubule formation in PaC. Beside, the nude mice tumor immunohistochemical staining showed fewer capillary-like vessels in POSTN-silenced group. Diagnostic imaging has significant clinical value and contrast-enhanced ultrasound (CEUS) has been increasingly used to evaluate tumor angiogenesis [19, 35]. The contrast agent SonoVue is a blood pool contrast agent [36] that can distribute entirely within the blood vessels and dynamically reveal the blood supply in the tumor mass and the microvessel structure in real time [37, 38]. We performed contrast-enhanced ultrasound on the subcutaneous tumors of mice before they were sacrificed. Consistent with the *in vitro* experiments, tumors in the control group showed high enhancement reflecting a rich blood flow signal, whereas reduced blood flow signals were observed in the POSTN knockdown group. Not surprisingly, the volume and weight of subcutaneous xenografts were decreased in nude mice derived from the POSTN knockdown group as insufficient nutrients were supplied for tumor growth. Along with these important clues, we further researched the potential association between POSTN expression and angiogenesis to better understand the mechanisms for how these processes could be regulated by POSTN.

As to blood supply of tumor, we turned our attention to the “classic” gene, such as VEGF. It is the most potent angiogenic factor for its high specificity to endothelial cells [33, 39, 40]. Our study demonstrated that increased POSTN expression significantly promoted Erk Phosphorylation and VEGF expression. Erk, affecting cellular angiogenesis, was detected in this study. Interestingly, we observed that Erk inhibitor SCH772984 inhibited Erk phosphorylation and significantly decreased VEGF expression as well as tubule formation of HUVECs in rPOSTN-treated PaC cells. In accordance with these observations, we found that knockdown of POSTN in PaC cells and tissues decreased Erk phosphorylation and its downstream VEGF expression. Additionally, the proliferation and migration of HUVECs were also decreased in SCH772984-treated group. Besides, in our study, we observed VEGF expression was decreased in POSTN-silenced group by nude mice tumor immunochemistry. Taken together, these findings suggested that POSTN promoted PaC angiogenesis, at least in part, via Erk signaling.

In conclusion, our findings elucidated the critical role of POSTN in PaC angiogenesis, POSTN could promote PaC metastasis and tumor angiogenesis via Erk/VEGFsignaling pathways, which may be serve as a marker in highly aggressive phenotype of PaC. In addition, inhibition of POSTN could suppress PaC progression *in vitro* and *in vivo*, suggesting that POSTN inhibition might represent new and potential strategies against human PaC. Collectively, we may identify a promising targeted therapy for PaC patients based on POSTN.

## MATERIALS AND METHODS

### Ethical statement

Informed consent was obtained from all participants and this research was approved by the ethics committee of Shanghai General Hospital affiliated of Shanghai Jiaotong University and performed in accordance with ethical principles. All mouse experiments were manipulated and housed according to the protocols approved by Shanghai Medical Experimental Animal Care Commission.

### Cell lines and reagent

The human PaC cell lines BxPC3 and SW1990 were purchased from American Type Culture Collection (Manassas, VA), Human umbilical vein endothelial cells (HUVECs) were purchased from Shanghai Institutes for Biological Sciences, Chinese Academy of Sciences. These cells were maintained in RPMI 1640 with 10% FBS. Human pancreatic stellate cells (PSCs) were purchased from ScienCell research laboratory (Carlsbad, CA) and maintained in stellate cell medium (ScienCell). All cells were cultured in a humidified atmosphere of 5% CO_2_ at 37°C. Human recombinant POSTN protein (rPOSTN) was purchased from Biovendor (Heidelberg, Germany) and dissolved in 0.1 M acetate buffer (pH 4) at a concentration of 1 μg/mL.

### Tissue microarray construction

PaC samples and paired adjacent non-tumor tissues with informed consent were collected from 30 patients who underwent pancreatic surgery and were stored at Biobank Center of National Engineering Center for Biochip at Shanghai. Tissue microarray was stained for expression analysis of POSTN (ab14041, Abcam, 1:50 dilution), VEGF (sc-152, Santa Cruz Biotechnology, 1:50 dilution). All immunohistochemically stained sections were dependently scored by two in-house pathologists who were blinded to clinical outcome.

### Lentivirus transduction for gene silencing

The lentivirus suspension used for shRNA silencing of the POSTN gene in PSCs was purchased from Ebioeasy Ltd (Shanghai, China). The target sequences for POSTN were 5′-CGGTGACAGTATAACAGTAAA-3′ named POSTN sh1, 5′-CACTTGTAAGAACTGGTATAA-3′ named POSTN sh2, respectively. The sequence for scrambled negative control shRNA was 5′-CCTAAGGTTAAGTCGCCCTCG-3′ named Control sh (Supplementary Figures 1). The PSCs lentivirus infection was performed according to the manufacturer's instructions.

### Endothelial tube formation assay

Briefly, each well of prechilled 96-well plates was covered with a thin layer of Matrigel, which was allowed to polymerize at 37°C for 1 h. HUVECs were resuspended in medium with different concentrations of rPOSTN. HUVECs (50 μl, 1 × 10^4^ cells/well) were added to the polymerized Matrigel. After incubation for 8 h at 37°C with 5% CO_2_, the tube formation ability was evaluated by measuring the tubular length and counting the number of tubes and tubular intersecting nodes in five random fields using Image Pro Plus software. Each experiment was performed at least three times.

### Cell proliferation assay

Cell proliferation was measured by Cell Counting Kit-8 (Dojindo, Kumamoto, Japan) according to the manufacturer's instructions. HUVECs (2×10^3^ cells/well) were cultured with different concentrations of rPOSTN in 96-well plates. The rPOSTN concentration was selected based on data shown in Supplementary Figures 2. Cell proliferation was examined every 24 h for 5 days after incubation for 2 h at the absorbance 450 nm.

### Cell migration and invasion assays

For cell migration and invasion assays, 6.5-mm Transwells^®^ chamber with 8.0-μm Pore Polycarbonate Membrane Insert (Corning, NY) was used according to the manufacturer's instructions. In invasion assay, diluted Matrigel (BD Biosciences) was plated in transwell chambers. HUVECs (4 × 10^4^) in 200 μL serum-free medium were added to the upper chamber and cultured for 48 h. In migration assay, the membranes were not coated with Matrigel and HUVECs (2 × 104) were cultured in the same condition. For both assays, 500 μl of medium with different concentrations of rPOSTN was added to the lower chamber as a chemoattractant. Finally, the migrated cells and invasive cells on the bottom of the membrane were fixed with 20% methanol and stained with 0.04% crystal violet and cells were photographed and counted under a microscope.

### Western blotting

Whole cell protein lysates were electrophoresed on 10% sodium dodecyl sulfate-polyacrylamide gels and transferred onto polyvinylidene difluoride membranes (Millipore). The membranes were blocked with 5% non-fat milk in Tris-buffered saline and then incubated with primary antibodies at 4°C overnight. The primary antibodies were as follows: anti-POSTN (1:1,000; ab14041, Abcam, Cambridge, UK), anti-Erk (1:1,000; #4695; Cell Signaling Technology), anti-P-Erk^Thr202/Tyr204^ (1:1,000; #9101; Cell Signaling Technology), anti-VEGF (1:1,000; sc-152; Santa Cruz Biotechnology) and anti-β-actin (1:5,000; Abcam). Membranes were then incubated with peroxidase-linked secondary antibody for 2 h at room temperature. Target protein signals were visualized by an enhanced chemiluminescence detection system (Amersham Bioscience, Piscataway, NJ, USA) according to the manufacturer's protocol. All western blotting analyses were performed three times.

### Nude mice tumorigenesis

SW1990 (3×10^6^ cells/mouse) and POSTN sh1 or control sh stably transfected PSCs (3×10^6^ cells/mouse) were subcutaneously co-injected into 4-week-old male nude mice (Institute of Zoology, Chinese Academy of Sciences, Shanghai, China). Tumor nodules were examined weekly, and were evaluated using the following formula: tumor volume = (Width^2^×Length)/2 [41]. Mice were killed 4 weeks after injection. Before sacrifice, contrast-enhanced ultrasound (CEUS) was performed using SonoVue as a contrast agent to assess angiogenesis *in vivo*. Lyophilized SonoVue powder was dissolved in 5 mL saline and 100 μL of suspension was injected via the post-glomus venous plexus within 2–3 s, followed by a 100 μL saline flush. Real-time observation of the xenograft was performed for at least 120 s. Dynamic images were preserved for later analysis. Finally, tumors were excised, weighed and fixed for immunohistochemistry staining.

### Immunohistochemistry

The paraffin-embedded nude mice tumor tissue was sliced into 4-μm thick sections and then deparaffinized and rehydrated. Immunohistochemical staining of sections was performed using primary antibody mouse anti-POSTN (1:100; Abcam, Cambridge, UK), primary antibody rabbit anti-VEGF (1:100; Santa Cruz Biotechnology), mouse anti-CD31 (1:100; Santa Cruz Biotechnology) at 37°C for 2 h. CD31 positive vessels indicated blood vessels in tissues. Finally, the analysis was assessed and scored by two independent investigators simultaneously by combining both the scores and staining intensity. Scores representing the percentage of positive staining tumor cells were categorized into: 0 (0–5%), 1 (6–25%), 2 (26–50%), 3 (51%–75%) and 4 (>75%) [42]. The intensity of staining was categorized into 0 (negative), 1 (weak, light yellow), 2 (moderate, yellow brown), or 3 (strong, brown) [43]. The final score was determined by the product of staining intensity and percentage of positive tumor cells (final score = intensity score × percentage score). The final score of ≤ 3 was considered as low expression, and > 3 as high expression [42].

### Statistical analysis

All statistical analyses were carried out using the SPSS 16.0 statistical software package. Statistical comparisons were conducted using Student's t-test and presented as mean ± SD. Correlation between POSTN and VEGF expression was analyzed by Spearman test. In all cases, *P*-value <0.05 was considered as statistical significant.

## SUPPLEMENTARY FIGURES


