# Supplementary material for: Periostin promotes tumor angiogenesis in pancreatic cancer via Erk/VEGF signaling
Source: Oncotarget. 2016 May 20;7(26):40148–59. doi: 10.18632/oncotarget.9512 (PMC5129999; doi:10.18632/oncotarget.9512)
Supplement: Supplementary file 1 [file oncotarget-07-40148-s001.pdf]

Periostin promotes tumor angiogenesis in pancreatic cancer via Erk/VEGF signaling

SUPPLEMENTARY FIGURES

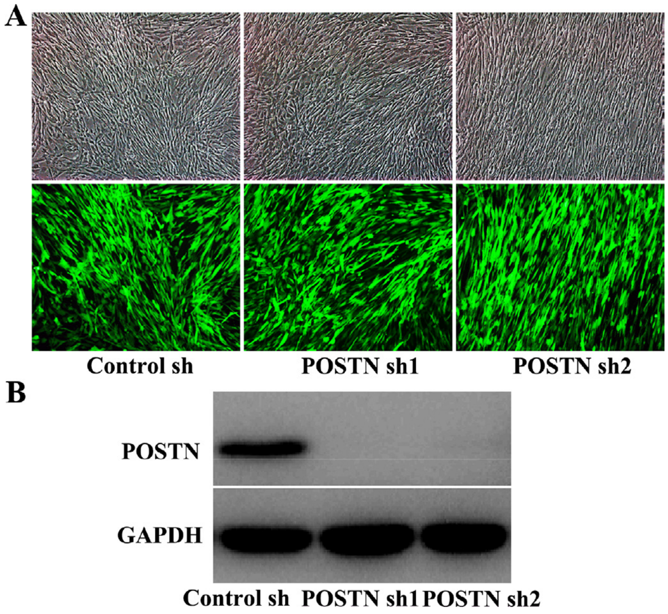

Supplementary Figure S1: Validation of lentiviral transfection efficiency for Control sh, POSTN sh1 and POSTN sh2 in pancreatic stellate cells (PSCs). A. PSCs were infected with lentiviral supernatant for 48 h and selected with 1 μg/mL puromycin to generate stable transfected cells. Positive cells expressing GFP fusion protein were photographed under fluorescence microscopy. **B.** POSTN expression levels in PSCs stably transfected with Control sh, POSTN sh1, or POSTN sh2 were confirmed by western blot analysis. GAPDH was used for normalization.

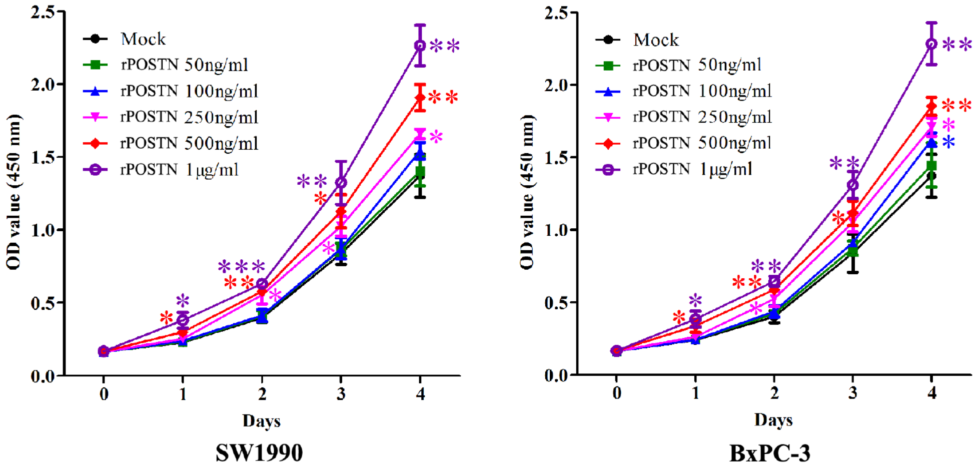

Supplementary Figure S2: Proliferation of SW1990 and BxPC-3 cells incubated with different concentrations of rPOSTN. Proliferation was measured by the CCK-8 assay. The proliferation rate of pancreatic cancer cells was highest with an rPOSTN concentration of 1 µg/mL and 500 ng/mL.
